# Comparative Study of the Water-Soluble Antioxidants in Fodder Additives and Sheep Blood Serum by Amperometric and Biochemical Methods

**DOI:** 10.3390/ani10071186

**Published:** 2020-07-13

**Authors:** Sergei Yu. Zaitsev, Anastasia A. Savina, Andrei A. Volnin, Oksana A. Voronina, Nadezhda V. Bogolyubova

**Affiliations:** L.K. Ernst Federal Science Center for Animal Husbandry, Dubrovitsy 60, Podolsk Municipal District, 142132 Moscow, Russia; kirablackfire@mail.ru (A.A.S.); volnin.a@mail.ru (A.A.V.); voroninaok-senia@inbox.ru (O.A.V.); 652202@mail.ru (N.V.B.)

**Keywords:** sheep blood serum, antioxidant activity, chitosan additives, high-protein concentrate, amperometric method

## Abstract

**Simple Summary:**

Feed additives are widely used to enrich animal diets in order to optimize digestive and metabolic processes. However, variations in the technology of preparation of even single-component compositions can play a significant role in the effects of their use. The effects of chitosan as a feed additive for animals on various digestive processes are important to study because of the animal nutrition and production quality, healthcare and farming, etc. We studied the antioxidant activity of various feed components: chitosan from various manufacturers and a high protein microbiologic synthesis concentrate by amperometric method. The antioxidant activity values in the rumen content and sheep blood were then examined and evaluated using these feed additives in the diet. The animal experiments were carried out on six rumen-fistulated ewes (in three groups). The particular tendencies of correlation value changes of the antioxidant activity vs. major biochemical parameters of a blood serum of rumen-fistulated ewes (in three groups) or some indicators of the rumen content were obtained for the first time. These data and correlations can be useful for understanding of the features of the sheep antioxidant status and for the evaluation of the feed additives and factors in sheep nutrition.

**Abstract:**

The effects of chitosan as feed additive for animals (FAFAs) on various digestive processes are an important to study because of the animal nutrition and production quality, healthcare and farming. The aims of this study were to evaluate the total amount of water-soluble antioxidants (TAWSA) of chitosan and high protein microbiologically synthesized concentrate as FAFAs; to assess the effect of these FAFAs on TAWSA values, parameters of sheep blood serum and rumen content by biochemical, physical and chemical methods. The laboratory studies of TAWSA values of feed components based on chitosan from different manufacturers or/and a high-protein concentrate were implemented. The animal experiments were carried out on six rumen-fistulated ewes (in three rounds of 14 days each, i.e., three groups) to confirm the results of the laboratory studies. The particular differences of the TAWSA of sheep blood by using both FAFAs by amperometric method were determined. A strong negative correlation −0.67 (or −0.86) was observed between TAWSA and the total protein (globulin’s) content in the blood for the Group 3 of animals. A moderate (0.40) or strong (0.73) positive correlation between TAWSA and total protein content in the blood for the Group 2 of animals than weak correlation 0.23 (or 0.26) for the control Group 1. In conclusion, the correlations between the value changes of TAWSA vs. major biochemical parameters of a blood serum of rumen-fistulated ewes (Group 3 > Group 2 > Group 1) or some indicators of the rumen content (ingesta pH, total content of volatile fatty acids, etc.) were found for the first time.

## 1. Introduction

The dietary beneficial properties of chitosan not only for humans, but also for monogastric and polygastric animals are widely known [1,2,3,4,5]. There are limited data on chitosan related to its impact on the total amount of water-soluble antioxidants (TAWSA) and biochemical parameters of sheep blood serum and rumen content for ewes, as well as no correlations between these parameters have been quantitatively described. The most interesting chitosan applications include those for human oral intake, animal dietary admission and compositions for external medicines. For example, in the extended review [6] claims that, in the case human intake “chitosan preparations result in a significantly greater weight loss; decrease in total cholesterol”; as well as “decrease in systolic and diastolic blood pressure compared with placebo”. The mechanism of chitosan action in animals and humans consist in molecular sorption and inhibition of some digestive enzymes [6,7,8]. The latest research “on chitosan based tissue engineering constructs, drug-delivery vehicles as well as dental care products” was summarized in the recent review [7]. In some cases, it is used as an integral component of wound healing tissues [7] and artificial skin due to its mucoadhesive properties [8], as part of “chondroprotective ointments” and biodegradable surgical material [9].

Such a profound diversity of chitosan applications is due to its useful properties such as biodegradability, bioavailability, nontoxicity, non-allergenicity, chelating and sorption properties [10]. Another interesting (though not widely reported) feature of chitosan is its antioxidant activity. Antioxidant properties of glucosamine (monomer unit of chitosan) differ to some extent for the cases of oligomeric, low and high molecular weight (polymeric) chitosan [9,10,11]. In the case of using chitosan as a feed additive for large and small ruminants, its major effects are due to its presence in the rumen, where numerous digestive processes occur (due to microbial fermentation) [8,9,10,11]. The major known changes in the biochemical parameters of the animal blood induced by chitosan usage are the following: ratio of the major volatile fatty acids (VFA), total fat, mineral composition [11].

In this context, the determination of the antioxidant properties of the polymeric and oligomeric chitosan as feed additive for healthy animals is an important but barely researched topic. However, the chemical properties and biological effects of chitosan exerted on the body strongly depend on the characteristics of the product, such as polydispersity, degree of deacetylation (DD) and molecular weight. Chitosan is a heteropolysaccharide in which at least 50% of the monomer units are of glucosamine [8,9,10,11]. The remainder, N-acetylglucosamine, stems from non-hydrolyzed chitin residues; therefore, the degree of deacetylation is an important parameter determining the chemical properties of chitosan [10]. In addition, partial destruction (i.e., main chain breaking) takes place during alkaline or acid hydrolysis of chitin [10]. Thus, depending on the accuracy and refinement of the manufacturing process, the product (chitosan) will have variations of the two major parameters (i.e., molecular weight and polydispersity). There is no doubt that chitosan samples with different chemical properties will have different effects on a living organism. In addition, dosage is also important when it comes to oral administration. On the other hand, chitosan is used as an adjuvant for oral and mucosal vaccines (by mechanically mixing chitosan with a protein), which improves the body’s immune response [12].

One can assume that mixing chitosan with feed protein may increase the effectiveness of the intake of both these components. Studies by Russian scientists have shown that the introduction of chitosan into the diet contributes to the better survival of young animals [13]. The positive effect of the chitosan usage (in combination with some probiotics) on the calf’s body is already known: an increase both in the level of humoral “immune defense” and in their total protein content in the animal blood serum [13]. There are certain cell protection mechanisms consisting of enzymes with oxidoreductase activity, non-enzymatic proteins, thiol-containing amino acids, polypeptides and other water- and fat-soluble substances against excess free radical production. In our opinion, the water-soluble substances (including amino acids and some proteins) have the most potent properties [14,15,16,17,18,19].

The aims of this study were to evaluate the total amount of water-soluble antioxidants (TAWSA) in feed additives based on chitosan (from different manufacturers) and high protein concentrate (through microbiologic synthesis) as FAFAs; to assess the effect of these FAFAs on TAWSA values, parameters of sheep blood serum and rumen content by biochemical, physical and chemical methods.

## 2. Materials and Methods

### 2.1. Feed Additives

The following feed additives were investigated: high protein microbiologic synthesis concentrate; chitosan 1 (deacetylation degree, DD, 83%, “Bioprogress” Ltd., Moscow region, Shchelkovo, Russia); chitosan 2 (DD 87%, “Qingdao Sinsur Chemical Co.” Ltd., Shandong, China) and their mixtures: M-1 or mixture 1) high-protein microbiologic synthesis concentrate mixed with chitosan 1 in a weight ratio of 1:1.5; M-2 or mixture 2) high protein microbiologic synthesis concentrate mixed with chitosan 2 in a weight ratio of 1:1.5. The samples M-1 and M-2 were prepared by mechanical mixing of the components at a temperature of 25 °C.

### 2.2. Rumen-Fistulated Ewes

**Figure 1:**
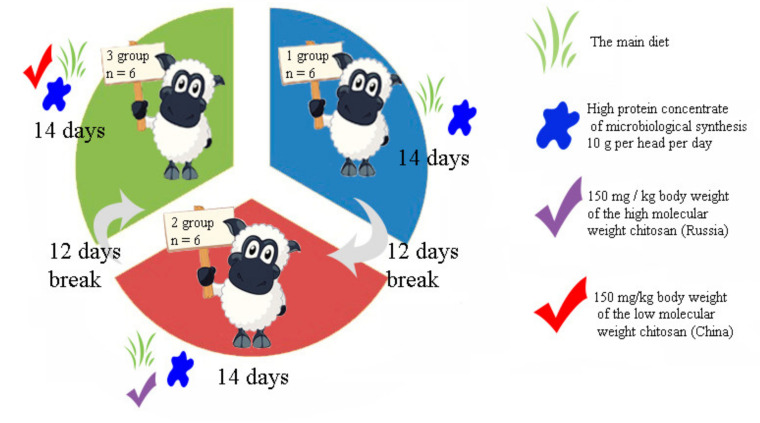
Schematic drawing of the experiments on six rumen-fistulated ewes.

The major experiments were carried out on 6 rumen-fistulated ewes only (in three rounds of 14 days each, i.e., three sheep groups with “permanent” fistula of the rumen) in order to reduce the number of animals used (Figure 1).

Group 1 was the control (the main diet only); Group 2 was the experimental one (the main diet and 150 mg per kg body weight of the high molecular weight chitosan 1, obtained from Russian company); Group 3 was the experimental group (the main diet and 150 mg per kg body weight of the low molecular weight chitosan 2, obtained from Chinese company). The main diet consisted of the standard nutrition and high protein concentrate of microbiologic synthesis 10 g per head per day (Table S1. Supplementary materials).

The experimental protocols concerning these three sheep groups were approved by the Scientific Council of the Federal Science Center for Animal Husbandry named after Academy Member L.K. Ernst (L.K. Ernst Federal Science Center for Animal Husbandry). All experiments and conditions (animal care, feeding, biologic material sampling, etc.) were performed in accordance with the applicable regulations (internationally recognized guidelines and STATEMENT of the Ethical Treatment of Animals Used in Research of the L.K. Ernst Federal Science Center for Animal Husbandry, Russia).

### 2.3. Animal Blood

Blood samples from all experimental animals were taken from the jugular vein (“vena jugularis”) 3 h after morning feeding using the vacuum system (Vacuette) into test tubes for clinical trials (Greiner Bio-One, Kremsmünster, Austria). The samples of the blood serum were taken from each animal (in the group) three to five times during the 14 days of each experiment. After partial retraction of the blood clot, the serum was separated by centrifugation for 15 min at 3000 rpm/min (laboratory centrifuge CM-12, Moscow, Russia). The biochemical indicators of animal blood serum sample were determined using a “ChemWell” automatic biochemical analyzer (Awareness Technology, Palm City, FL, USA) with reagents of “Analyticon Biotechnologies AG” (Lichtenfels, Germany) and “Spinreact” (Barcelona, Spain).

The following biochemical indicators: the concentration of total protein (biuret method), albumin (colorimetric method), urea (enzymatic colorimetric Berthelot method), creatinine (the kinetic Yaffe method), glucose (enzymatic glucose oxidase method), cholesterol (enzyme-colorimetric method), phospholipids (enzyme-colorimetric method), bilirubin (quantification by Walters and Gerarde method); calcium (O-cresolphthalein complexone method), phosphorus, magnesium and iron (colorimetric method); alanine aminotransferase (ALT) activity (UV-kinetic method), aspartate aminotransferase (AST) activity (UV-kinetic method), alkaline phosphatase activity (kinetic method) were determined [15,19,20,21,22,23,24,25]. The following ratios and indicators: A/G, Ca/P, ALT/AST and the concentration of globulins were determined by calculation [15,19].

### 2.4. Samples of Rumen Content

Samples of rumen content were also taken from ewes 3 h after morning feeding through a rumen fistula using a Janet syringe. These samples were taken from each animal (in the group) three to five times during the 14 days of each experiment. The following indicators were determined in the rumen content [19,26,27,28,29,30]: (1) rumen pH on the Aquilon 410 instrument; (2) total volatile fatty acids (VFA) by steam distillation on a Margham apparatus; (3) the concentration of ammonia nitrogen by the microdiffusion method according to Conway; (4) amylolytic activity using the photometric method using a KFK-3-01-ZOMZ spectrophotometer–photometer device (Russia); (5) the amount of biomass of protozoa and bacteria by the method of differential centrifugation on a Sigma 3–18 centrifuge (SIGMA Laborzentrifugen GmbH, Osterode am Harz, Germany).

### 2.5. Sample Preparations

The samples for the study of antioxidant activity were prepared as follows: 0.2 g of the high protein concentrate was placed in a 200 mL beaker; 25 mL of double-distilled water were poured; the mixture was intensively stirred with a glass rod and put on a shaker for 60 min (rotation speed 120 rpm); after sample extraction, the coarse sediment was filtered through a paper filter (“FILTRAK”, soft broad-pore filtering quickly for coarse precipitation); the filtrate was adjusted to 50 mL in a volumetric flask and stirred for 10 min.

The polymer of Chinese origin (chitosan 2) has slightly swelled, while the chitosan produced by “Bioprogress” (chitosan 1) has precipitated to a greater extent. chitosan was partially dissolved in the same double-distilled water as the high-protein concentrate. The coarse precipitate (water-insoluble fraction) was separated by filtering through a 10-times-fold folded sterile gauze followed by centrifugation at total speed of 3000 rpm for 10 min. The resulting solution was clear and free of the above mentioned suspension. The supernatant was diluted in 50 mL of eluent and selected for analysis.

The serum samples for the study of antioxidant activity were prepared as follows: blood serum was gently, without foaming, stirred for a minute; 100 μL was taken and injected into 2 mL of double-distilled water in a flask. The study the total antioxidant activity of the prepared samples (mentioned above) was performed by amperometric method. The measurements were carried out on the “Tsvet-Yauza 01-AA” instrument. The total concentration of water-soluble antioxidants was determined by measuring the electric current generated during the oxidation of molecules at the surface of the working electrode (at a certain potential applied). The mass fraction of water-soluble antioxidants was measured and represented in equivalents of gallic acid. The calibration solutions (0.2; 0.5; 1 and 4 mg/L, respectively) were prepared by dilution of the “stock solution” of gallic acid (100 mg/L). A trend line was drawn from the calibration graph and a linear equation is calculated using “Microsoft Excel” software. The mass concentration of water-soluble antioxidants in the samples is calculated using a calibration graph plotted (the peak area vs. the output signal), which varies with the concentration of gallic acid [14]. As an eluent, a solution of phosphoric acid 0.0022 mmol/L is used (at room temperature 22 °C, humidity ca. 81%).

### 2.6. TAWSA Calculations and Correlations

The calculation of the TAWSA mass concentration (X, mg/g) was carried out according to the formula [14]:

X = (X_G_·N·V_n_)/(m_n_·1000) (1)

where

X_G_ is the mass concentration of antioxidants found according to the calibration graph, mg/L;
N is the dilution ratio of the analyzed sample;
V_n_ is the volume of the solution (extract) of the analyzed sample, mL;
m_n_ is the amount of the analyzed sample, g.

The correlations between the total content of TAWSA and major biochemical parameters of a blood serum of rumen-fistulated ewes (the concentration of total protein and its major fractions, carbohydrates, lipids, minerals, nitrogen metabolites, as well as the activity of enzymes in the ewe’s blood for each of the animal groups) or some indicators of the rumen content (ingesta pH, total content of volatile fatty acids, etc.) were calculated using statistical treatment by STATISTICA 6.0 (the average errors were below 1%). The correlations were divided into the following quartiles:

±0.76–±1.0 = very strong positive (negative) correlations;
±0.51–±0.75 = strong positive (negative) correlations;
±0.26–±0.50 = moderate positive (negative) correlations;
±0.01–±0.25 = weak positive (negative) correlations.

## 3. Results

### 3.1. TAWSA Parameters of Chitosan Feed Additives and High Protein Concentrate

The measurements of the total amount of water-soluble antioxidants (TAWSA) of feed additives based on chitosan (from different manufacturers) and high protein concentrate of microbiologic synthesis were the first part of the presented research. It is important to highlight that the addition of both types of chitosan led to a visual changes in the obtained solutions. The filtrate of the high-protein concentrate was rather turbid, while the filtrates of the mixture with chitosan were almost transparent, especially in the case of the mixture M-2 (i.e., with chitosan 2). The differences in the “antioxidant activities” of the control and the samples can be illustrated by the following parameters (Table 1): (i) **S**, the average peak area on the originally measured amperometric curve; (ii) **y**, the TAWSA value calculated from the calibration curve; (iii) **x**, the TAWSA value calculated by the Equation (1).

The measured “antioxidant activity” of chitosan 2 sample was almost twice higher compared to chitosan 1 sample (Table 1). The additional experiments have shown that the amount of the water-soluble part in the chitosan 2 sample was almost twice higher compared to chitosan 1 water-soluble part. Thus, recalculated (“real”) TAWSA values for the water-soluble parts of chitosan 1 and 2 samples were almost the same.

### 3.2. TAWSA Parameters of the Blood Serum of Rumen-Fistulated Ewes

The major animal experiments were carried out on 6 ewes at 3 rounds (each round for these ewes was exactly 14 days as described in detail in the “Materials and methods” section) to clarify the results of laboratory studies. The average TAWSA values measured in the serum samples of all sheep groups were in the range of 20.4–21.9 mg/g (Table 2).

### 3.3. The Major Biochemical Parameters of the Blood Serum of Rumen-fistulated Ewes

The major biochemical parameters of the blood serum of fistula ewes are summarized in Table 3. These parameters were stable throughout the experiments with some minor deviations in the Groups 2 and 3 as discussed in Part 4.

### 3.4. The Major Parameters of a Rumen Fluid of Rumen-Fistulated Ewes

The rumen fluid is a liquid suspension in the ruminant’s stomach where swallowed food enters. The major parameters of a rumen fluid of rumen-fistulated ewes are presented in Table 4.

An increase in the content of volatile fatty acids (VFA) in the rumen of Group 3 animals by 29.5% was found to be associated with a decrease in the pH of the rumen content. In all studied groups of animals, amylolytic activity was at the level of 19.20–21.30 U/mL, and the concentration of ammonia was at the level of 16.26–17.84 mg/dL. These and other parameters of a rumen fluid of fistula ewes are discussed in Part 4.

### 3.5. The Correlations between the TAWSA and the Major Biochemical Parameters of a Blood Serum of Rumen-Fistulated Ewes

Finally, we have calculated the correlation coefficients between the total content of TAWSA and the concentration of total protein and its major fractions, carbohydrates, lipids, minerals, nitrogen metabolites, as well as the activity of enzymes in the ewe’s blood for each of the animal groups (Table 5).

A strong negative correlation (−0.67) was observed between TAWSA and the total protein content in the blood for the Group 3 of animals (Table 5). A moderate positive correlation (0.40) between TAWSA and total protein content in the blood for the Group 2 of animals than weak correlation (0.23) for the control Group 1 (Table 5). This tendency of correlation value changes of TAWSA vs. total blood protein between the studied groups (Group 3 > Group 2 > Group1) was even more pronounced in the case of the globulin’s content in the ewe’s blood: a very strong negative correlation (−0.86) was found for the animal Group 3 and strong positive correlation (0.73)—for the animal Group 2 than weak correlation (0.26) for the control Group 1 (Table 5).

There were no clear dependences between TAWSA and glucose content in the ewe’s blood for all three groups regardless of diet (Table 5).

### 3.6. The Correlations between the TAWSA and the Rumen Content of Rumen-Fistulated Ewes

The next task of our research was to determine the correlation dependencies between TAWSA in the blood and some indicators of the rumen content (ingesta pH, total content of volatile fatty acids, etc.) (Table 6).

These comparative parameters were determined in the blood samples and rumen ingesta content, taken 3 h after the beginning of feeding, respectively. These results will be discussed below.

## 4. Discussion

In the study of biochemical parameters characterizing protein metabolism in animals, a tendency toward an increase in urea concentration in experimental groups of animals was noted. This trend points to an increase in the processes of protein metabolism for sheep, especially for animals in the experimental groups. Creatinine content in sheep blood increased by 13.0% in the experimental Group 3 and by 38.3% in the experimental Group 2 (*p* < 0.05) compared to the control group. This growth correlates with increased metabolism in the body, which requires the mobilization of energy resources. It is known that the main function of creatine phosphate is to ensure the stability of the production of intracellular energy by constantly maintaining the required level of adenosine triphosphoric acid (ATP) by its resynthesis [15].

Comparison of TAWSA and indicators of nitrogen metabolism revealed that there was a number of correlations of varying degrees between the studied parameters. It was shown in our previous studies [19,25] that strong or very strong correlations were the most informative than moderate ones (no matter, positive or negative by sign). In contrast, no visible tendency was observed for the correlation values of TAWSA vs. albumins to globulins ratio (A/G), probably, because of the clear differences in albumins content in the ewe’s blood (Table 5). It was very important that such a tendency “worked” perfectly in the case of correlations between TAWSA and urea content in the ewe’s blood (Table 5).

A significant increase in ALT activity by 54% (*p* < 0.05) when using M-2 mixture in animal nutrition can be explained by the activation of protein metabolism in the body of these animals. It is known that the biochemical role of aminotransferases consists in the transfer of amino groups from amino acids to keto-containing acids [15].

Studying the parameters of rumen metabolism revealed a tendency to increase a VFA content in the sheep rumen when chitosan was used in the animal nutrition, which indicated an increase in enzymatic processes in the animal rumen, especially in the cases of both experimental groups.

A strong negative correlations between TAWSA and the concentration of low molecular weight urea in animal serum for all three groups were observed regardless of diet. Moreover, these negative correlation coefficients between TAWSA and urea were 7.7% and 23.1% higher after chitosan 1 or 2 addition to the diet (Groups 2 and 3, respectively) than the control Group 1 (Table 5). Urea, synthesized in the liver and in the rumen wall from ammonia nitrogen, amino acids and amides, is the end product of nitrogen metabolism for all ruminants, including ewes [15,16,17]. Urea is usually accounted for at least half of the animal residual nitrogen in the blood and 80%–83% of the urine [15,16,17]. There are interesting notes about the correlations between blood urea changes and protein metabolism for cattle (supporting our abovementioned findings for sheep): “a decrease in blood urea indicates a more efficient use of amino acids of the exchange protein on protein synthesis” [31]; “… a decrease in the level of urea nitrogen in the blood serum indicates a more efficient assimilation of the nitrogen of the diet as a whole” [32]. In addition, it is known that urea is also a representative of the class of low molecular weight antioxidants [18,19,20]. The opposite tendency of correlation between TAWSA and creatinine was observed between the studied groups (Group 1 > Group 2 > Group3). Moderate and strong negative correlations were found for the Groups 3 and 2, which were about 41.6% and 22.1% less than very strong negative correlations (−0.77) found for the control Group 1 (Table 5). The creatinine content in the blood of sheep increased by 13.0% in experimental Group 3 and by 38.3% in experimental Group 2 (*p* < 0.05) than the control group. Since the main function of creatine phosphate is to ensure the availability of intracellular energy by constantly maintaining the required level of adenosine triphosphoric acid (ATP) through its resynthesis [15], it can be assumed that the use of chitosan positively affected the energy metabolism in the animal body. In contrast, the tendency of correlation between TAWSA and some lipids (like triglycerides and cholesterol) between the studied groups (Group 3 > Group 2 > Group 1) was the same as for protein and nitrogen metabolism (Table 5). For example, in the case of triglycerides, a very strong and strong negative correlations were found for the Groups 3 and 2, which were about 78.7% and 34.0% higher (in absolute values) than moderate positive correlation (0.47) found for the control Group 1 (Table 5). In the case of cholesterol, a moderate negative and positive correlations were found for the Groups 3 and 2, which were about 146.7% and 80.0% higher (in absolute values) than weak positive correlation (0.15) found for the control Group 1 (Table 5). This was a trend (even comparing the groups by absolute differences in percentages) in spite of the huge differences in absolute correlation values as shown for lipids. Similar relative changes between the studied Groups 3 and 2 that were found about 33.3% or 37.0% (between these two groups in the case of triglycerides or cholesterol, respectively). Hence, the similarity in these relative changes (in percentages) between the studied groups 3 and 2 (both for triglycerides and cholesterol) can be definitely due to the ewe’s intake of different chitosan samples as diet additives. This effect can be explained by the significant difference in the water-soluble parts of two chitosans: chitosan 2 (used for the Group 3 nutrition) has had much more content of the water-soluble part than chitosan 1 (used for the Group 2 nutrition). This tendency was supported by our data [21,22,23,24,25] concerning the regulatory effects of chitosan on the activity of lipolytic enzymes, mainly pancreatic lipase in the gastrointestinal tract.

Unexpectedly, there was no similar tendency found (Table 5) in the case of the studied enzymes (ALT, AST, alkaline phosphatase). The only strong positive correlation between TAWSA and the enzyme activity of alkaline phosphatase (0.68) was found (Table 5).

In the case of the mineral metabolites, strong positive correlations between TAWSA and iron content in the Groups 1 (0.69), 2 (0.74) and 3 (0.58) also deserve special attention (Table 5). In addition, strong negative correlations between TAWSA and chloride content in the Groups 1 (0.67), 2 (0.65) and 3 (0.57) were found (Table 5).

The moderate and strong positive correlations (0.38 and 0.77, respectively) were found between TAWSA and rumen acidity in Groups 1 and 2, respectively (Table 6). It is well-known that ingesta pH values of the rumen contents depend on the particular diet and they can be slightly acidic or slightly alkaline, which is the most important condition for the development of microorganisms and for the biochemical processes in the pancreas to occur [15]. The pH values affect all aspects of the functioning of the ruminant’s ingesta and the animal body as a whole [33,34,35]. Acidification of the ruminant’s ingesta (i.e., a decrease in pH) negatively affects the enzymatic activity of some microorganisms [33]. The main consequence of lowering pH below 6.0 is the decrease of cellulolytic activity of rumen bacteria [34], which is associated with a decrease in the ability to maintain pH inside bacteria cells at such a low pH. Moreover, pH values of rumen’s content below 5.0 were detrimental to ciliary protozoa [35].

The very strong (0.82) and moderate (0.33) positive correlations were noted between TAWSA and ammonia nitrogen in the animal rumen for the Groups 3 and 1, respectively (Table 6). It is known that ammonia nitrogen is the main nitrogen source for microbial growth and synthesis of microbial proteins in the animal rumen [36]. An increase in its content in the animal rumen can lead to a more intensive growth of microbial mass, which is a valuable source of essential amino acids for the host organism. Moreover, amino acids and urea play important roles in the body’s antioxidant defense system [15,19]. The authors considered the changes in the concentration of ammonia (in the animal rumen) are associated with an increase in TAWSA in the ewe’s blood.

It is worth noting that there was no reliable correlation between TAWSA and the amylolytic activity or VFAs formation in the animal rumen (Table 6). It was clear that both the VFA’s formation and their absorption, which vary in the rate with different acids, occurred in the animal rumen [15]. Correlation analysis allowed us to conclude that there was a moderate negative correlation (−0.32) between the content of VFA in the rumen and TAWSA in the blood serum of the Group 3 (Table 6). A different picture was observed when high-molecular chitosan was used in the ewe’s diet together with protein feed. There was a moderate correlation (0.42) between the studied indicators in the blood serum of Group 2 (Table 6). Apparently, the biologic properties of chitosan influenced these relationships. In recent years, a fairly large number of studies were carried out showing that VFAs were really potent modulators of the activity of the macroorganism’s immune system. It is necessary to continue research in the direction of assessing the activity of individual classes of antioxidants to clarify the mechanisms of the found effects and formation of ewe’s antioxidant status in details.

## 5. Conclusions

The numerous novel data on sheep nutrition using chitosan, antioxidant activities, blood biochemistry and rumen microbiology were obtained, the major ones: the correlation coefficients between TAWSA and the major biochemical parameters of the sheep blood, as well as between TAWSA and the major indicators of their rumen content. The particular tendencies of correlation value changes of TAWSA vs. majority of the biochemical parameters of a blood serum of rumen-fistulated ewes (Group 3 > Group 2 > Group 1) were found. The opposite tendency of correlation value changes of TASWA vs. creatinine content was observed between the studied groups (Group 1 > Group 2 > Group 3). These data and correlations were obtained for the first time and can be useful for understanding of the features of the sheep antioxidant status and for the evaluation of the feed additives and factors in sheep nutrition. In the future, the authors intend to perform a study of the combined feed additives based on a wider range of different chitosans with the aim of elucidating more detailed correlations of their effects on the major antioxidant defense systems of the Romanovskaya breed ewes, which is one of the most important for Russian livestock.

## Figures and Tables

**Table 1:** Total amount of water-soluble antioxidants (TAWSA) in absolute values and calculated according to the gallic acid standard.

Sample	S Peaksa a.u.	y mg/L	x mg/g
Chitosan 1	476	0.186	0.047
Chitosan 2	939 **	0.325 **	0.081 **
High-protein concentrate	8028	2.553	0.638
M-1, i.e., chitosan 1 & high-protein concentrate	5080	1.668	0.834
M-2, i.e., chitosan 2 & high-protein concentrate	5419 *	1.770 *	0.885 *

Notes: *—*p* < 0.05; **—*p* < 0.01—differences are statistically significant within the experimental period compared with the Group 1 (control).

**Table 2:** TAWSA measured in the sheep serum samples—absolute and average values.

Group 1			Group 2			Group 3		
Sample №	S Peaks, a.u.	x, mg/g	Sample №	S Peaks, a.u.	x, mg/g	Sample №	S Peaks, a.u.	x, mg/g
1	2677	23.2	7	2256	19.8	13	2423	21.2
2	2357	20.6	8	2459	21.5	14	1356	12.6
3	2200	19.4	9	2918	25.1	15	3250	27.8
4	2542	22.1	10	2210	19.5	16	2580	22.4
5	2149	19.0	11	2244	19.7	17	2936	25.3
6	2046	18.2	12	1941	17.3	18	2502	21.8
Average values		20.4			20.5			21.9 *

Notes: *—*p* < 0.05; **—*p* < 0.01—differences are statistically significant within the experimental period compared with the Group 1 (control).

**Table 3:** Major biochemical parameters of a sheep serum.

Parameters	Groups					
	1 (Control)		2 (Exp. M-1)		3 (Exp. M-2)	
	M	m	M	m	M	m
Total protein, g/L	80.77	1.77	83.44	1.62	77.38	1.14
Albumins, g/L	37.08	1.08	35.89	0.83	36.23	1.05
Globulins, g/L	43.69	1.51	47.54	1.82	41.15	0.62
Albumins/Globulins	0.85	0.04	0.76	0.04	0.88	0.03
Urea, mM/L	5.51	0.61	7.71	0.58	6.37	0.39
Creatinine, µM/L	68.40	5.08	94.59 *	7.41	77.29	7.29
Glucose, mM/L	2.70	0.11	1.27	0.20	2.34	0.15
Triglycerides, mM/L	0.28	0.01	0.27	0.01	0.28	0.01
Cholesterol, mM/L	1.66	0.12	1.71	0.13	1.47	0.05
Alanine aminotransferase, U/L	12.94	1.06	8.38	1.74	19.93 **	1.08
Aspartate aminotransferase, U/L	62.85	3.53	66.86	3.55	67.11	5.31
Alkaline phosphatase, U/L	135.95	23.80	126.40	17.74	101.91	17.61
Calcium, mM/L	2.40	0.18	2.36	0.16	2.51	0.15
Phosphorus, mM/L	2.06	0.23	2.18	0.23	2.55	0.26
Magnesium, mM/L	1.04	0.06	0.96	0.05	0.94	0.02
Iron, µM/L	27.78	1.97	25.73	2.06	27.41	1.54
Chlorine, mM/L	117.08	1.94	118.59	1.87	111.17	1.16

Notes: *—*p* < 0.05; **—*p* < 0.01—differences are statistically significant within the experimental period compared with the Group 1 (control).

**Table 4:** The major parameters (M ± m) of a rumen fluid of rumen-fistulated ewes.

Parameters	Group 1		Group 2		Group 3	
	M	m	M	m	M	m
pH, a.u.	6.88	0.08	6.96	0.09	6.57	0.10
VFA, a.u.	7.33	0.23	7.75	0.32	9.49	0.37
Amylolytic activity, U/mL	21.30	0.84	19.20	1.28	19.74	0.85
Ammonia, mg/dL	16.89	0.47	16.26	0.35	17.84	0.26
Infusoria (I), a.u.	0.45	0.07	0.37	0.05	0.34	0.07
Bacteria (B), a.u.	0.30	0.05	0.25	0.03	0.23	0.05
Total (I&B), a.u.	0.75	0.07	0.62	0.05	0.57	0.07

Notes: *—*p* < 0.05; **—*p* < 0.01—differences are statistically significant within the experimental period compared with the Group 1 (control).

**Table 5:** Correlation coefficients between the TAWSA and the major biochemical parameters of a blood serum of rumen-fistulated ewes.

Total Amount of Water-Soluble Antioxidants	Group 1	Group 2	Group 3 *
Total protein, g/L	0.23	0.40	−0.67 **
Albumins, g/L	0.01	−0.81 **	−0.16
Globulins, g/L	0.26	0.73 **	−0.86 **
Albumins/globulins	−0.78 **	−0.85 **	0.25
Urea, mM/L	−0.78 **	−0.84 **	−0.96
Creatinine, µM/L	−0.77 **	−0.60 *	−0.45 *
Glucose, mM/L	−0.50 *	0.04	−0.39
Triglycerides, mM/L	0.47 *	−0.67 **	−0.84 **
Cholesterol, mM/L	0.15	0.27	−0.37
Alanine aminotransferase, U/L	0.40	−0.53 *	0.07
Aspartate aminotransferase, U/L	−0.47 *	−0.72 **	0.01
Alkaline phosphatase, U/L	0.68 **	0.38	−0.34
Calcium, mM/L	0.62 **	0.06	−0.32
Phosphorus, mM/L	−0.33	0.18	0.26
Magnesium, mM/L	−0.07	0.02	−0.56 *
Iron, µM/L	0.69 **	0.74 **	0.58 *
Chlorine, mM/L	−0.67 **	−0.65 **	−0.57 *

Notes: *—*p* < 0.05; **—*p* < 0.01—differences are statistically significant.

**Table 6:** Correlation coefficients between the TAWSA (determined in the blood samples) and rumen content (taken 3 h after the beginning of feeding) of rumen-fistulated ewes.

TAWSA	Ingesta pH	Ammonia Nitrogen	Amylolytic Activity	Total VFA
Group 1	0.38	0.33	−0.27	−0.25
Group 2	0.77 *	0.04	0.10	0.42 *
Group 3	−0.01	0.82 **	−0.04	−0.32

Notes: *—*p* < 0.05; **—*p* < 0.01—differences are statistically significant.

## Supplementary Materials

The following are available online at https://www.mdpi.com/2076-2615/10/7/1186/s1, Table S1: Diet and composition of the standard compounds for sheep nutrition.
