# Supplementary material for: Comparative Study of the Water-Soluble Antioxidants in Fodder Additives and Sheep Blood Serum by Amperometric and Biochemical Methods
Source: Animals (Basel). 2020 Jul 13;10(7):1186. doi: 10.3390/ani10071186 (PMC7401643; doi:10.3390/ani10071186)
Supplement: Supplementary file 1 [file animals-10-01186-s001.pdf]

Supplementary materials to MS ID: animals-812259

Diet nutrition:
Metabolic energy, MJ = 13.2
Crude protein, g = 180.0
Crude fat, g = 40.0
Crude fiber, g = 380.0

The composition of the standard compound feed for sheep:
Metabolic energy, MJ / kg = 10.5
Crude protein,% = 17.0
Starch + sugar,% = 40.0
Crude fiber,% = 12.0
Crude fat,% = 2.5
Ca,% = 0.5-0.6
P,% = 0.7 - 0.8
NaCl,% = 0.80-1.0
Moisture,% = 14.0
Premix contains zinc, copper, selenium, sulfur, iodine, vitamins A, E, D.

The chemical composition of the protein of microbiological synthesis in % to dry matter:
moisture = 10.1 ± 2.0;
ash = 9.0 ± 2.2;
protein = 47.2+ 1.4;
carbohydrates = 21.0 ± 3.2;
lipids = 8.1 ± 2.0.

Technological requirements and quality of protein:
protein not less than 47.2 ± 1.4,%
including amino acids:
lysine-7.0 ± 2.1;
arginine - 2.7 ± 1.1;
histidine - 2.1 ± 0.4;
glycine - 4.1 ± 1.2;
valine - 6.9 ± 0.5;
alanine - 9.0 ± 2.1;
leucine - 6.2 ± 1.9;
isoleucine - 4.6 ± 1.5;
proline - 2.8 ± 1.8:
serine - 3.2 ± 1.4;
methionine - 1.6 ± 0.7;
threonine -2.6- ± 0.8;
tryptophan -1.2 ± 0.6;
phenylalanine-2.1 ± 1.3;
tyrosine - 2.0 ± 0.6;
cysteine - 1.1- ± 0.8;
ornithine-1.2 ± 0.4.
